# Coagulopathy Induced by Veno-Arterial Extracorporeal Membrane Oxygenation Is Associated With a Poor Outcome in Patients With Out-of-Hospital Cardiac Arrest

**DOI:** 10.3389/fmed.2021.651832

**Published:** 2021-04-30

**Authors:** Takumi Tsuchida, Takeshi Wada, Satoshi Gando

**Affiliations:** ^1^Division of Acute and Critical Care Medicine, Department of Anesthesiology and Critical Care Medicine, Hokkaido University Faculty of Medicine, Sapporo, Japan; ^2^Department of Acute and Critical Care Center, Sapporo Higashi Tokushukai Hospital, Sapporo, Japan

**Keywords:** veno-arterial extracorporeal membrane oxygenation, out-of-hospital cardiac arrest, post-cardiac arrest syndrome, disseminated intravascular coagulation, antithrombin

## Abstract

**Background:** In recent years, the use of veno-arterial extracorporeal membrane oxygenation (VA-ECMO) in patients with cardiopulmonary arrest who do not respond to conventional resuscitation, has increased. However, despite the development of VA-ECMO, the outcomes of resuscitated patients remain poor. The poor prognosis may be attributed to deterioration owing to the post-cardiac arrest syndrome (PCAS); this includes the systemic inflammatory response and coagulation activation caused by the extracorporeal circulation (VA-ECMO circuit) itself. This study aimed to evaluate the coagulofibrinolytic changes caused by VA-ECMO and to identify predictive factors of poor prognosis.

**Methods:** We analyzed 151 cases of PCAS with witnessed cardiac arrest. As biomarkers, platelet counts, prothrombin time ratio, fibrin/fibrinogen degradation products, fibrinogen, antithrombin, and lactate were recorded from blood samples from the time of delivery to the third day of hospitalization. The maximum (max) and minimum (min) values of each factor during the study period were calculated. To evaluate the impact of VA-ECMO on patients with PCAS, we performed propensity score matching between the patients who received and did not receive VA-ECMO. Sub-analysis was performed for the group with VA-ECMO.

**Results:** There were significant differences in all baseline characteristics and demographics except the time from detection to hospital arrival, percentage of cardiopulmonary resuscitations (CPR) by witnesses, and the initial rhythm between the groups. Propensity score matching adjusted for prehospital factors demonstrated that the patients who received VA-ECMO developed significantly severe coagulation disorders. In a sub-analysis, significant differences were noted in the prothrombin time ratio min, fibrinogen max, antithrombin max, and lactate min between survivors and non-survivors. In particular, the prothrombin time ratio min and antithrombin max were strongly correlated with poor outcome.

**Conclusion:** In the present study, significant coagulopathy was observed in patients who received VA-ECMO for CPR. In particular, in patients receiving VA-ECMO, the minimum prothrombin time ratio and maximum antithrombin by day 3 of hospitalization were strongly correlated with poor outcomes. These results suggest that VA-ECMO-induced coagulopathy can be a promising therapeutic target for patients resuscitated by VA-ECMO.

## Introduction

Patients resuscitated after out-of-hospital cardiac arrest (OHCA) often develop a post-cardiac arrest syndrome (PCAS), a complex combination of the pathophysiological processes of post-cardiac arrest brain injury, post-cardiac arrest myocardial dysfunction, systemic ischemia/reperfusion responses, and persistent precipitating pathology (1). The main pathophysiology of the systemic ischemia-reperfusion response is the systemic inflammatory response syndrome (SIRS) and hypercoagulation, that lead to disseminated intravascular coagulation (DIC) (2, 3). DIC is characterized by the widespread activation of tissue-factor-dependent coagulation, inadequate control of coagulation by physiological anticoagulation pathways due to endothelial activation and damage, fibrin formation within the vessels, and eventually thrombotic occlusion of the vessels and associated deterioration of oxygen supply to cells and tissues (4). These changes cause damage to the microvasculature and organ dysfunction (4, 5). In particular, DIC-induced thrombotic obstruction of the brain, called the “no-reflow phenomenon,” is characterized by impaired reperfusion after cerebral ischemia, despite stable systemic circulatory status (6). These findings suggest that PCAS-related coagulopathy is closely associated with the pathophysiology of post-cardiac arrest brain injury, which is the leading cause of death in patients with PCAS (2).

In recent years, guidelines suggest the use of veno-arterial extracorporeal membrane oxygenation (VA-ECMO) in patients with cardiopulmonary arrest (CPA) who are unresponsive to conventional resuscitation (7, 8); in addition, substantial experience and research data have been accumulated on VA-ECMO as a resuscitative strategy. Although the recommendation for VA-ECMO in the guideline is of level of 2b (level of evidence C-LD [limited data]) (8), the survival rate and neurological outcomes of PCAS remain poor.

A recent study showed that extracorporeal circulation itself induces systemic inflammation and coagulation activation due to the exposure of the patient's blood to non-endothelialized surfaces of the ECMO circuit (9). These results suggest that the induction of VA-ECMO exacerbates PCAS-related SIRS and DIC, leading to poor outcomes in those who receive VA-ECMO. Nonetheless, the prognostic effect of VA-ECMO-induced coagulopathy remains unclear.

The purpose of this study was to evaluate the coagulofibrinolytic changes caused by ECMO and to identify factors that may be associated with poor outcomes.

## Materials and Methods

### Patients

We identified 246 patients aged 18 years or older, who were resuscitated after OHCA due to cardiac causes from January 2010 to December 2017, and were subsequently admitted to the intensive care unit of the Hokkaido University Hospital. The exclusion criteria were as follows: (1) patients under 18 years of age, (2) those who were not resuscitated after cardiac arrest, (3) those who had cardiac arrest due to trauma, acute aortic dissection, or rupture of aortic aneurysms, (4) those on anticoagulant therapy, and (5) those with an underlying coagulofibrinolytic disorder. After excluding cases with unknown times of cardiac arrest and cases with missing data, we analyzed data from those with witnessed cardiac arrest. We retrospectively conducted a systematic review of the computer-based medical records of these patients to obtain baseline data and DIC-related parameters. Coagulofibrinolytic markers, including platelet counts, prothrombin time (PT) ratios, fibrin/fibrinogen degradation products (FDP), fibrinogen values, antithrombin values (AT), and lactate values from the blood samples, were recorded from the time of arrival to the third day of hospitalization. Each parameter was measured at the following four time points: time of arrival in the ED, ED arrival to 24 h after hospitalization, 24–48 h after hospitalization, and 48–72 h after hospitalization. To evaluate the changes in each factor on the prognosis, the maximum (max) and minimum (min) values of each factor at these four points were also calculated. All the patients were divided into two groups: the VA-ECMO+ group, consisting of patients who received VA-ECMO and the VA-ECMO- group, consisting of patients who did not receive VA-ECMO. VA-ECMO was introduced in accordance with the criteria of the SAVE-J study (Table 1) (10). Unfractionated heparin was used for anticoagulation during VA-ECMO. The dose of unfractionated heparin was adjusted to maintain an activated clotting time (ACT) of 180–220 seconds or an activated partial thromboplastin time (APTT) of 1.5–2.5 times the baseline value.

**Table 1:** Criteria for implementing veno-arterial extracorporeal membrane oxygenation.

Inclusion criteria
VF/VT on the initial ECG
No ROSC at least during the 15 min after hospital arrival (or after contact with a doctor) even though conventional CPR was performed
Within 45 min from reception of the emergency call or the onset of cardiac arrest to the hospital arrival
Exclusion criteria
Under the age of 20 years or those aged 75 years or older
Poor level of activities of daily livings before the onset of cardiac arrest
No informed consent from the individuals representing patients

*VF, ventricular fibrillation; VT, ventricular tachycardia; ECG, electrocardiogram; ROSC, return of spontaneous circulation; CPR, cardiopulmonary resuscitation*.

### Definition

DIC was diagnosed based on the Japanese Association for Acute Medicine (JAAM) DIC score (11) and the International Society on Thrombosis and Haemostasis (ISTH) DIC score (5). Organ failure was assessed using the Sequential Organ Failure Assessment (SOFA) score (12). In addition, during the study period, we calculated the maximum and minimum values for each score as well as those for the coagulofibrinolytic markers. Disease severity was assessed according to the Acute Physiology and Chronic Health Evaluation (APACHE) II score (13). The outcomes were assessed using the hospital mortality and cerebral performance category (CPC) scale (14) at 28-hospital days. We defined CPC 1 and 2 as favorable neurological outcomes and CPC 3 to 5 as unfavorable neurological outcomes.

### Statistical Analysis

Data for continuous variables have been presented as medians and interquartile ranges (25th–75th percentiles). Categorical data have been presented as frequencies and percentages. Patient characteristics and outcomes were compared between the two groups using the Mann–Whitney U test (for numerical variables) and the Fisher's exact test (for categorical variables). Propensity score matching was performed between the VA-ECMO+ group and the VA-ECMO- group using three factors: age, sex, and time from detection to hospital arrival. A sub-analysis evaluated the impact of each coagulofibrinolytic marker on the prognosis using logistic regression analysis (backward elimination [likelihood ratio]) and area under the curve (AUC) of the receiver operating characteristic (ROC) curve. The optimal cutoff value of the ROC curve was calculated using the Youden index. Variables found to be statistically significant at the 10% level on univariate analysis were included in the multivariate model. All analyses were performed using IBM SPSS software (version 25; IBM Japan, Tokyo, Japan). All reported *p* values were two-tailed, and differences were considered statistically significant at *p* values of < 0.05.

### Ethics

The study protocol was approved by our Institutional Review Board (approval number: 180831), and the requirement for informed consent was waived due to the retrospective design of the study.

## Results

### Clinical Characteristics of the Patients

**Figure 1:**
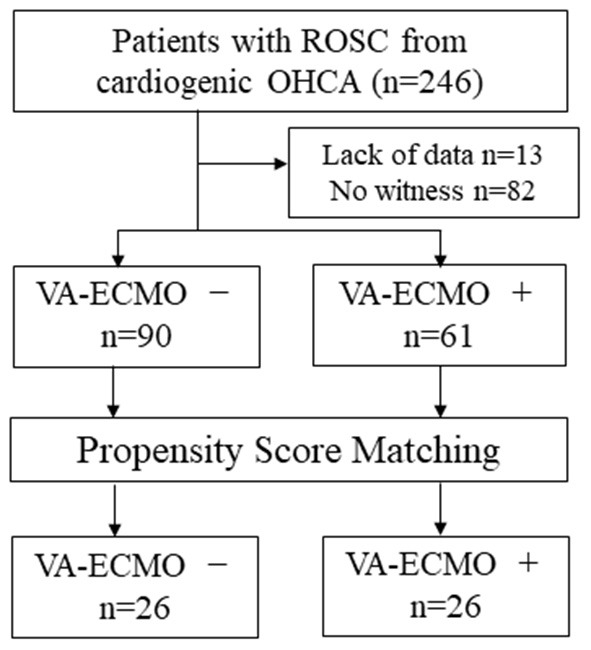
Flow chart of study population. ROSC, return of spontaneous circulation; OHCA, out-of-hospital cardiac arrest; VA-ECMO, veno-arterial extracorporeal membrane oxygenation.

We analyzed 151 cases from 246 OHCA patients with ROSC, excluding those with no witnesses and those with missing data (Figure 1). Table 2 shows the background of the OHCA patients (*n* = 151) who were included in this study. Sixty-one patients received VA-ECMO (VA-ECMO+) and 90 did not (VA-ECMO-). There was a significantly higher proportion of males in the VA-ECMO+ group. In addition, the VA-ECMO+ group was younger than the VA-ECMO- group. Prehospital factors, including the interval between the receipt of the emergency call and hospital arrival, cardiopulmonary resuscitation (CPR) by witness and shockable rhythm did not differ significantly between VA-ECMO+ and VA-ECMO- groups. However, the time interval between the receipt of the emergency call and the commencement of the ROSC or VA-ECMO in the VA-ECMO+ group was much longer than that in the VA-ECMO- group. In addition, the APACHE II, DIC, and SOFA scores in the VA-ECMO+ group were significantly higher than those in the VA-ECMO- group, and both the neurological outcomes and hospital mortality were significantly worse in the VA-ECMO+ group than in the VA-ECMO- group.

**Table 2:** Baseline characteristics of witnessed out-of-hospital cardiac arrest.

	**VA-ECMO −** **(*n* = 90)**	**VA-ECMO +** **(*n* = 61)**	***p*-Value**
Age, yr	73.5 (61.0–80.0)	63.0 (52.0–68.0)	<0.001
Gender; male (%)	54 (60.0)	53 (88.3)	<0.001
Time interval (min)			
1	33.0 (27.0–38.0)	31.0 (27.0–44.0)	0.780
2	25.0 (16.3–37.0)	51.0 (42.0–64.0)	<0.001
CPR by witness, n (%)	54 (60.0)	37 (60.7)	0.536
Shockable rhythm, n (%)	43 (47.8)	40 (65.6)	0.072
Adrenalin dosage (mg)	0.0 (0.0–1.0)	2.0 (1.0–3.0)	<0.001
APACHE II score	33.0 (28.0–36.0)	37.0 (34.0–41.0)	<0.001
JAAM DIC score max	3.0 (2.0–5.0)	7.0 (6.0–8.0)	<0.001
JAAM DIC score min	1.0 (0.0–1.8)	5.0 (3.0–6.0)	<0.001
ISTH DIC score max	3.0 (2.0–4.0)	6.0 (5.0–6.0)	<0.001
ISTH DIC score min	0.0 (0.0–2.0)	3.0 (1.0–4.0)	<0.001
SOFA score max	7.0 (6.0–10.0)	13.0 (12.0–15.0)	<0.001
SOFA score min	3.0 (1.3–5.0)	10.0 (7.0–12.0)	<0.001
Hospital stay	13.5 (5.0–21.8)	12.0 (5.0–24.0)	0.824
Unfavorable neurological outcome (n, %)	61 (67.8)	54 (88.5)	0.003
Hospital mortality (n, %)	18 (20.0)	30 (49.2)	<0.001

*Data presented as median (25th−75th percentile), percentage or numbers*.

*1, interval between the receipt of the emergency call and the hospital arrival; 2, interval between the receipt of the emergency call and ROSC or ECMO start; ROSC, return of spontaneous circulation; VA-ECMO, veno-arterial extracorporeal membrane oxygenation; CPR, cardiopulmonary resuscitation; APACHE II, Acute Physiology and Chronic Health Evaluation II; JAAM, Japanese Association for Acute Medicine; ISTH, International Society on Thrombosis and Haemostasis; SOFA, Sequential Organ Failure Assessment; maximum, max; minimum, min*.

*The unfavorable neurological outcome is defined by the cerebral performance category (CPC) scale as 3 to 5 at 28-hospital day*.

### Propensity Score Matching Adjusted for Prehospital Factors

In the comparison of the VA-ECMO+ group with the VA-ECMO- group, propensity score matching was performed between the two groups for the three factors: age, sex, and time from detection to hospital arrival; this was performed to reduce any bias due to individual patient variations and the level of severity before arrival at the hospital. The propensity score model had a c-statistic of 0.885, which indicated good discrimination between the two groups. Using the propensity score matching process, 26 patients were ultimately selected from each group. Table 3 shows the characteristics of the matched patients. There were no significant differences in the time factors before arrival at the hospital between the two groups, and there was no difference in the time from cardiac arrest to ROSC. The APACHE II scores tended to be higher in the VA-ECMO+ group, but were not significantly different; the two groups generally had balanced characteristics. The VA-ECMO+ group had worse scores for each item that comprehensively assessed the severity of organ failure and DIC. Furthermore, significant severe coagulopathies were found in the VA-ECMO+ group (Table 4). The VA-ECMO+ group showed a significant increase in fibrinolytic markers and a decrease in platelets, fibrinogen, and AT. There was no significant difference in hospital mortality between the two groups, but there was a trend toward poorer outcomes in the VA-ECMO+ group. Even after adjusting for prehospital factors by propensity score matching, the VA-ECMO+ group had higher SOFA, ISTH DIC, and JAAM DIC scores, and more severe coagulopathy than the VA-ECMO- group.

**Table 3:** Baseline characteristics of witnessed out-of-hospital cardiac arrest after propensity score matching.

	**VA-ECMO −** **(*n* = 26)**	**VA-ECMO +** **(*n* = 26)**	***p*-Value**
Age, yr	62.5 (55.3–76.0)	66.5 (58.5–72.8)	0.905
Gender; male, n (%)	21 (80.1)	22 (84.6)	0.500
Time interval (min)			
1	34.5 (27.3–39.3)	30.0 (27.0–38.3)	0.436
2	34.0 (21.0–44.0)	42.0 (29.0–53.0)	0.245
CPR by witness, n (%)	15 (57.7)	16 (61.5)	0.500
Shockable rhythm, n (%)	21 (80.8)	19 (73.1)	0.372
Adrenalin dosage (mg)	0.0 (0.0–1.6)	2.0 (1.3–3.8)	<0.001
APACHE II score	34 (29.5–36.0)	37 (33.0–39.8)	0.060
JAAM DIC score max	3.5 (2.0–5.0)	7.0 (6.0–8.0)	<0.001
JAAM DIC score min	1.0 (1.0–1.0)	5.0 (3.3–6.0)	<0.001
ISTH DIC score max	3.0 (2.0–4.0)	5.5 (5.0–6.0)	<0.001
ISTH DIC score min	0.0 (0.0–1.8)	3.5 (1.3–4.0)	<0.001
SOFA score max	7.0 (5.3–8.0)	13.0 (11.3–14.8)	<0.001
SOFA score min	3.0 (2.0–4.8)	10.0 (6.0–12.0)	<0.001
Hospital stay	16.5 (8.5–28.8)	11.0 (4.3–24.8)	0.216
Unfavorable neurological outcome (n, %)	18 (69.2)	22 (84.6)	0.066
Hospital mortality (n, %)	5 (19.2)	11 (42.3)	0.143

*Data presented as median (25th−75th percentile), percentage or numbers*.

*1, interval between the receipt of the emergency call and the hospital arrival; 2, interval between the receipt of the emergency call and ROSC or ECMO start; ROSC, return of spontaneous circulation; VA-ECMO, veno-arterial extracorporeal membrane oxygenation; CPR, cardiopulmonary resuscitation; APACHE II, Acute Physiology and Chronic Health Evaluation II; JAAM, Japanese Association for Acute Medicine; ISTH, International Society on Thrombosis and Haemostasis; SOFA, Sequential Organ Failure Assessment; maximum, max; minimum, min*.

*The unfavorable neurological outcome is defined by the cerebral performance category (CPC) scale as 3 to 5 at 28-hospital day*.

**Table 4:** Results of coagulation and fibrinolysis markers and lactate levels in propensity score matched patients.

	**VA-ECMO –** **(*n* = 26)**	**VA-ECMO +** **(*n* = 26)**	***p*-Value**
Platelet counts on day 0 (10^9^/L)	169 (132–216)	75 (57–103)	<0.001
Platelet counts max (10^9^/*L*)	169 (132–216)	88 (71–106)	<0.001
Platelet counts min (10^9^/L)	12.5 (9.4–14.8)	5.5 (4.7–7.7)	<0.001
PT ratio on day 0	1.1 (1.0–1.3)	1.7 (1.4–2.2)	<0.001
PT ratio max	1.2 (1.1–1.4)	1.8 (1.4–2.3)	<0.001
PT ratio min	1.0 (1.0–1.1)	1.1 (1.0–1.3)	0.056
FDP on day 0 (mg/L)	23.4 (11.2–48.4)	205.0 (82.1–412.0)	<0.001
FDP max (mg/L)	24.5 (11.7–48.4)	205.0 (86.3–412.0)	<0.001
FDP min (mg/L)	5.9 (3.8–9.1)	16.8 (8.7–47.6)	<0.001
Fibrinogen on day 0 (g/L)	2.58 (2.12–3.01)	2.13 (1.50–2.47)	<0.001
Fibrinogen max (g/L)	4.87 (4.11–5.64)	3.97 (3.01–4.49)	0.001
Fibrinogen min (g/L)	2.45 (2.12–3.01)	2.08 (1.48–2.40)	0.004
AT on day 0 (%)	70.5 (61.0–85.3)	49.0 (42.0–63.8)	<0.001
AT max (%)	82.0 (69.0–90.5)	64.0 (53.0–70.8)	0.001
AT min (%)	68.0 (56.8–79.0)	44.0 (40.0–53.0)	<0.001
Lactate on day 0 (mmol/L)	10.0 (7.2–12.8)	15.0 (12.9–17.8)	<0.001
Lactate max (mmol/L)	10.0 (7.2–12.8)	15.0 (12.9–17.8)	<0.001
Lactate min (mmol/L)	1.0 (0.8–1.6)	1.7 (1.2–3.2)	0.001

*Data presented as median (25th−75th percentile)*.

*VA-ECMO, veno-arterial extracorporeal membrane oxygenation; PT, prothrombin time; FDP, fibrin/fibrinogen degradation products; AT, antithrombin*.

### Subgroup Analyses of the VA-ECMO Patients

**Figure 2:**
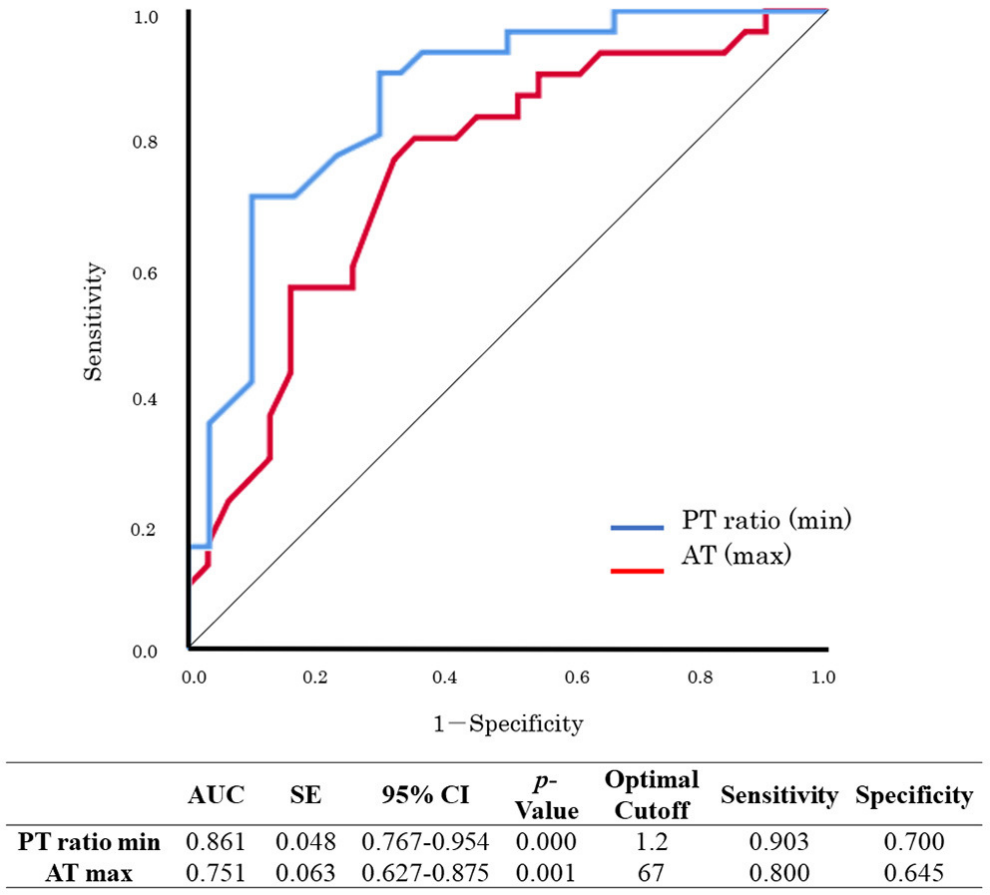
Receiver operating characteristic (ROC) curve analysis for hospital mortality. PT, prothrombin time; AT, antithrombin; maximum, max; minimum, min.; AUC, area under the curve; SE, standard error; CI, confidence interval.

Sub-analyses were performed on the data of 61 patients who received VA-ECMO. The patients were divided into two groups according to survival during hospitalization and neurological outcomes at day 28 after hospital admission (Tables 5, 6). There were no significant differences in pre-hospitalization factors between the two groups, even after grouping by outcome; however, there were significant differences in the SOFA scores (max), PT ratios (min), and AT values (max) in both analyses. Furthermore, there were significant differences in the SOFA scores (min), FDP values (max), fibrinogen values (max), and lactate values (min) between the survivors and non-survivors. Logistic regression analysis was also performed to evaluate the impact of each factor on mortality in patients receiving VA-ECMO (Table 7). Univariate analysis showed significant differences in the PT ratios (min), fibrinogen values (max), AT values (max), and lactate values (min). Multiple logistic regression analysis showed that the PT ratio (min) was an independent predictor of mortality during hospitalization (*p* = 0.009), and that AT (max) may be a predictor of mortality during hospitalization (*p* = 0.094). Figure 2 shows the results of the analysis for predicting hospital mortality using ROC curves for both factors. Both, the PT ratio (min) and the AT (max) were found to be good predictors of hospital mortality (AUCs of 0.861 and 0.751, respectively). The optimal cutoff value was 67 % (sensitivity: 80.0, specificity: 64.5) for AT (max) and 1.2 (sensitivity: 90.3, specificity: 70.0) for the PT ratio (min). Moreover, logistic regression analysis using ISTH DIC score components (PT ratio, platelet, FDP and fibrinogen) as independent variables showed that the PT ratio (min) was an independent predictor for hospital mortality (*p* = 0.001).

**Table 5:** Background characteristics and illness severities in patients who received VA-ECMO.

	**Survivors** **(*n* = 31)**	**Non-survivors** **(*n* = 30)**	***p*-Value**	**Favorable** **(*n* = 7)**	**Poor** **(*n* = 54)**	***p*-Value**
Age, yr	63.0 (48.0–68.0)	62.5 (53.0–69.8)	0.644	66.0 (47.0–67.5)	62.5 (52.3–68.8)	0.851
Gender; male, n (%)	27 (87.1)	26 (86.7)	0.628	6 (85.7)	47 (87.0)	0.647
Time interval (min)						
1	30.0 (27.0–42.0)	32.5 (27.3–43.3)	0.634	28.0 (25.5–34.0)	32.0 (27.0–44.0)	0.279
2	52.0 (44.0–66.5)	48.0 (38.0–61.8)	0.462	51.0 (48.5–56.5)	50.5 (39.8–64.0)	0.956
CPR by witness, n (%)	19 (61.3)	18 (60.0)	0.563	6 (85.7)	31 (57.4)	0.151
Shockable rhythm, n (%)	22 (71.0)	18 (60.0)	0.206	6 (85.7)	34 (63.0)	0.247
Adrenalin dosage (mg)	2.0 (0.0–3.0)	2.0 (1.3–3.0)	0.475	2.0 (1.5–2.5)	2.0 (1.0–3.0)	0.903
APACHE II score	37.0 (33.5–38.5)	38.0 (35.0–42.0)	0.107	36.0 (33.5–37.5)	38.0 (34.0–41.0)	0.428
JAAM DIC score max	8.0 (6.5–8.0)	7.0 (6.0–8.0)	0.160	7.0 (7.0–8.0)	7.5 (6.0–8.0)	0.682
JAAM DIC score min	4.0 (3.0–5.0)	5.0 (3.3–6.0)	0.725	4.0 (4.0–4.5)	5.0 (3.0–6.0)	0.634
ISTH DIC score max	6.0 (5.0–6.5)	6.0 (5.0–6.0)	0.638	6.0 (5.0–6.0)	6.0 (5.0–6.0)	0.974
ISTH DIC score min	3.0 (1.0–4.0)	4.0 (2.0–5.0)	0.098	2.0 (1.0–3.0)	4.0 (1.3–4.0)	0.161
SOFA score max	13.0 (12.0–14.0)	14.5 (12.0–17.0)	0.036	12.0 (10.5–12.5)	13.0 (12.0–15.0)	0.030
SOFA score min	9.0 (5.5–11.0)	11.0 (9.0–12.0)	0.015	8.0 (5.0–10.5)	10.0 (7.3–12.0)	0.250

*Data presented as median (25th−75th percentile), percentage or numbers*.

*1, interval between the receipt of the emergency call and the hospital arrival; 2, interval between the receipt of the emergency call and ROSC or ECMO start; ROSC, return of spontaneous circulation; VA-ECMO, veno-arterial extracorporeal membrane oxygenation; CPR, cardiopulmonary resuscitation; APACHE II, Acute Physiology and Chronic Health Evaluation II; JAAM, Japanese Association for Acute Medicine; ISTH, International Society on Thrombosis and Haemostasis; SOFA, Sequential Organ Failure Assessment; maximum, max; minimum, min*.

*Patient survival and death were evaluated during hospitalization*.

*The unfavorable neurological outcome is defined by the cerebral performance category (CPC) scale 3 to 5 at 28-hospital day*.

**Table 6:** Results of coagulation and fibrinolysis markers and lactate levels in patients receiving VA-ECMO.

	**Survivor** **(*n* = 31)**	**Non-survivor** **(*n* = 30)**	***p*-Value**	**Favorable** **(*n* = 7)**	**Poor** **(*n* = 54)**	***p*-Value**
Platelet counts max (10^9^/L)	89 (73–115)	90 (65–112)	0.702	89 (82–102)	87 (67–114)	0.765
Platelet counts min (10^9^/L)	52 (40–62)	46 (32–93)	0.614	54 (44–59)	50 (35–65)	0.765
PT ratio max ×10	1.8 (1.4–2.3)	1.8 (1.5–2.7)	0.480	1.8 (1.3–2.0)	1.8 (1.5–2.5)	0.310
PT ratio min ×10	1.1 (1.0–1.1)	1.4 (1.1–1.8)	<0.001	1.0 (1.0–1.1)	1.1 (1.0–1.5)	0.025
FDP max (mg/L)	258.0 (163.0–448.0)	135.3 (73.9–363.0)	0.091	310.0 (218.8–396.5)	200.0 (83.7–436.0)	0.232
FDP min (mg/L)	13.6 (8.1–24.5)	24.1 (13.6–53.9)	0.020	9.7 (7.7–18.8)	17.3 (12.0–48.2)	0.088
Fibrinogen max (g/L)	4.19 (3.71–4.72)	3.26 (2.48–4.77)	0.016	4.36 (3.96–4.48)	3.91 (2.96–4.79)	0.269
Fibrinogen min (g/L)	2.02 (1.51–2.32)	1.78 (1.46–2.37)	0.634	1.85 (1.37–1.98)	1.92 (1.49–2.35)	0.417
AT max (%)	70.0 (63.0–81.5)	57.5 (50.3–64.0)	0.001	98.0 (69.0–104.0)	64.0 (52.0–72.8)	0.002
AT min (%)	48.0 (38.0–57.5)	45.0 (38.0–55.5)	0.415	48.0 (40.0–58.0)	46.0 (38.0–56.0)	0.666
Lactate max (mmol/L)	15.0 (13.0–17.0)	16.5 (13.5–19.0)	0.193	15.0 (14.7–16.5)	15.5 (12.8–18.0)	0.715
Lactate min (mmol/L)	1.6 (1.3–2.3)	3.9 (2.0–14.4)	<0.001	1.5 (1.4–1.9)	2.3 (1.5–6.2)	0.088

*Data presented as median (25th−75th percentile)*.

*VA-ECMO, veno-arterial extracorporeal membrane oxygenation; PT, prothrombin time; FDP, fibrin/fibrinogen degradation products; AT, antithrombin; maximum, max; minimum, min*.

*Patient survival and death were evaluated during hospitalization*.

*The unfavorable neurological outcome is defined by the cerebral performance category (CPC) scale 3 to 5 at 28-hospital day*.

**Table 7:** The results of the univariate and multivariate logistic regression analysis for prediction of hospital mortality in patients receiving VA-ECMO.

**Variables**	**Univariate**			**Multivariate**		
	**Odds ratio**	**95% CI**	***p*-Value**	**Odds ratio**	**95% CI**	***p*-Value**
Age	1.009	0.971–1.049	0.644			
Gender	1.038	0.235–4.593	0.960			
Platelet counts max	0.988	0.864–1.131	0.865			
Platelet counts min	1.098	0.926–1.303	0.282			
PT ratio max	1.025	0.975–1.076	0.333			
PT ratio min	1.892	1.267–2.826	0.002	1.714	1.147–2.562	0.009
FDP max	0.998	0.996–1.001	0.168			
FDP min	1.013	0.997–1.030	0.121			
Fibrinogen max	0.996	0.992–0.999	0.025			
Fibrinogen min	0.996	0.998–1.005	0.393			
AT max	0.940	0.902–0.979	0.003	0.961	0.916–1.007	0.094
AT min	0.265	0.945–1.016	0.265			
Lactate max	1.111	0.966–1.278	0.139			
Lactate min	2.072	1.132–3.793	0.018			

*Data presented as median (25th−75th percentile)*.

*VA-ECMO, veno-arterial extracorporeal membrane oxygenation; PT, prothrombin time; FDP, fibrin/fibrinogen degradation products; AT, antithrombin; maximum, max; minimum, min*.

## Discussion

In the present study, more severe coagulopathy was observed in patients who received VA-ECMO for CPR than in those who did not receive VA-ECMO. This finding indicated that VA-ECMO may evoke coagulopathy itself. In the analysis of patients receiving VA-ECMO, there was a correlation between poor outcomes and PT ratios (min), fibrinogen values (max), AT values (max), and lactate values (min), with a particularly strong correlation between the PT ratio (min) and AT (max); this showed significant predictive ability for hospital mortality. These factors were also correlated with neurological outcomes. To the best of our knowledge, this was the first study to investigate coagulofibrinolytic responses associated with VA-ECMO.

Patients who received VA-ECMO experienced significantly more severe coagulopathy than those who did not receive VA-ECMO. However, the interval between the receipt of the emergency call and the commencement of ROSC or VA-ECMO in the VA-ECMO+ group was much longer than that in the VA-ECMO- group (Table 2). A previous study indicated that the degree of hypoxia, defined as the time from the onset of cardiac arrest to first CPR, and the duration of CPR were significant determinants of the severity of coagulopathy associated with PCAS (3, 15, 16). The current study showed that the patients who received VA-ECMO had significantly more severe coagulopathy than those who did not receive VA-ECMO, even after adjusting for the cardiac arrest time. These results indicate that VA-ECMO may itself cause the deterioration of coagulopathy associated with PCAS; this suggests that coagulofibrinolytic impairment induced by VA-ECMO may result in poor outcomes in patients with PCAS.

Logistic regression analysis showed that PT ratios (min) and AT values (max) were strongly correlated with poor outcomes in patients in the VA-ECMO+ group (Table 6 and Figure 2). The AT, which forms a complex with thrombin and inhibits thrombin and activated coagulation factor X, is an important anticoagulant factor. The Japanese Clinical Practice Guidelines for Management of Sepsis and Septic Shock recommend AT replacement therapy in patients with sepsis-associated DIC, whose AT activity has decreased to <70% (17). This is similar to the optimal cutoff value of 67 obtained in this study. Previous studies have confirmed reduced AT levels in patients with PCAS (18, 19), especially in those with DIC (20). In addition, it has been suggested that AT protects against myocardial ischemia and reperfusion injury (21). Although no prospective studies have shown the beneficial effects of AT therapy in cardiac arrest patients receiving VA-ECMO (22), these findings suggest that patients receiving VA-ECMO should be administered AT with a target of ~70% AT activity.

The main pathophysiology of DIC involves massive thrombin generation caused by tissue factor-factor VII pathway activation; this was formerly known as the extrinsic coagulation pathway, and its activity was generally assessed by PT. The current study showed coagulopathy with predominant prolongation of PT in the ECMO+ group. All patients who received VA-ECMO were administered unfractionated heparin for anticoagulation during the procedure. The dose of heparin, which was strictly adjusted in accordance to ACT or APTT, does not generally affect the value of PT. Patients with a PT ratio >1.2 have been shown to have a significantly higher incidence of mortality and multiple organ failure (23), and the JAAM DIC criteria awards 1 point with a PT ratio >1.2 (11); this is similar to the results obtained in the present study, that showed that the optimal cutoff value of the PT ratio for predicting hospital mortality in PCAS patients with VA-ECMO was 1.2.

Conversely, there were no significant relationships between DIC scores and outcomes (Table 5). The discrepancy of PT and DIC scores can be explained by the results of the logistic regression analysis, which showed that among the ISTH DIC score components, only the PT ratio was an independent predictor of hospital mortality. This finding suggests that in the ISTH DIC score, items other than PT do not sensitively reflect the outcome of PCAS patients, as these items may be affected by various factors that are not closely related to outcome.

PCAS is often compared to “sepsis-like syndrome,” which is commonly associated with high levels of circulating cytokines and coagulofibrinolytic abnormalities, namely DIC (24). The development of DIC has been recognized as one of the most critical conditions in sepsis due to its frequency and high severity. Previous studies have demonstrated that the mortality rate of sepsis patients with DIC was significantly higher than the overall mortality rate of sepsis patients (25, 26). In addition, a recent study showed that active screening and diagnosis of DIC in sepsis was associated with the improvement in patients' outcomes (27). This evidence may support our present findings, which suggest that the induction of VA-ECMO may itself cause the deterioration of DIC associated with PCAS. The evaluation of coagulation-related biomarkers, especially PT and AT, may predict poor outcomes in patients with PCAS who were resuscitated using VA-ECMO. In addition, VA-ECMO-induced coagulopathy can be a promising therapeutic target in these patients, and target values of AT and the PT ratio may be 70% and 1.2, respectively.

### Study Limitations

In the present study, 13 patients who received VA-ECMO and 29 patients who did not receive VA-ECMO died by day 3, resulting in a survival bias. This study was conducted retrospectively in a single institution, and the number of enrolled patients was relatively small. In addition, there may have been the potential for a selection bias and confounding due to unknown or unmeasured variables. Patients in this study did not receive platelet concentrates, AT, and fibrinogen products during the data collection period; however, the use of heparin, fresh frozen plasma, and other drugs that affect coagulation and fibrinolysis were not assessed.

## Conclusions

In the present study, significantly severe coagulopathy was observed in patients who received VA-ECMO for CPR. In particular, in patients receiving VA-ECMO, the minimum PT ratio and maximum antithrombin by day 3 of hospitalization had a strong correlation with poor outcomes. VA-ECMO-induced coagulopathy can be a promising therapeutic target for patients resuscitated using VA-ECMO.

## Data Availability Statement

The raw data supporting the conclusions of this article will be made available by the authors, without undue reservation.

## Ethics Statement

The studies involving human participants were reviewed and approved by Hokkaido University Hospital Division of Clinical Research Administration. Written informed consent to participate in this study was provided by the participants' legal guardian/next of kin.

## Author Contributions

TT and TW contributed to study conception, analysis, and manuscript preparation. SG contributed to manuscript preparation and revision for intellectual content. All authors read and approved the final version of the manuscript prior to submission.

## Conflict of Interest

The authors declare that the research was conducted in the absence of any commercial or financial relationships that could be construed as a potential conflict of interest.
